# Prebiotic Organic Microstructures

**DOI:** 10.1007/s11084-012-9290-5

**Published:** 2012-08-11

**Authors:** Marie-Paule Bassez, Yoshinori Takano, Kensei Kobayashi

**Affiliations:** 1 Département Chimie, Université de Strasbourg, 72 route du Rhin, 67400 Illkirch, France; 2 Institute of Biogeosciences, Japan Agency for Marine-Earth Science and Technology (JAMSTEC), 2-15 Natsushima, Yokosuka, 237-0061 Japan; 3 Department of Chemistry and Biotechnology, Yokohama National University, 79-5 Tokiwadai, Hodogaya-ku Yokohama, 240-8501 Japan

**Keywords:** Origin of life, Prebiotic chemistry, Prebiotic signatures, Geochemistry, Analytical chemistry

## Abstract

Micro- and sub-micrometer spheres, tubules and fiber-filament soft structures have been synthesized in our experiments conducted with 3 MeV proton irradiations of a mixture of simple inorganic constituents, CO, N_2_ and H_2_O. We analysed the irradiation products, with scanning electron microscopy (SEM) and atomic force microscopy (AFM). These laboratory organic structures produced a wide variety of proteinaceous and non-proteinaceous amino acids after HCl hydrolysis. The enantiomer analysis for D,L-alanine confirmed that the amino acids were abiotically synthesized during the laboratory experiment. We discuss the presence of CO_2_ and the production of H_2_ during exothermic processes of serpentinization and consequently we discuss the production of hydrothermal CO in a ferromagnesian silicate mineral environment. We also discuss the low intensity of the Earth’s magnetic field during the Paleoarchaean Era and consequently we conclude that excitation sources arising from cosmic radiation were much more abundant during this Era. We then show that our laboratory prebiotic microstructures might be synthesized during the Archaean Eon, as a product of the serpentinization process of the rocks and of their mineral contents.

## Introduction

Experiments simulating the primitive Earth atmosphere were conducted on gaseous mixtures of CO, N_2_/NH_3_ above liquid water irradiated with protons, helium ions, electrons, heavy ions, gamma and X and UV -rays, in a glass tube. Most of them led to proteinous and nonproteinous amino acids (Kobayashi et al. 2008). The first Kobayashi experiment irradiating with protons a gaseous mixture of CO/(CO+CO_2_) and N_2_ over liquid H_2_O was performed in 1989 (Kobayashi et al. 1989, 1990). The resulting liquid aqueous solution was filtered through a membrane filter (pore size: 0.2 μm). The analysis of the remainder of the solution led to amino acids. Mixtures of CO/(CO+CO_2_), N_2,_ H_2_O irradiated with 3 and 40 MeV protons, with 65 MeV helium nuclei and 400 MeV electrons, also produce amino acids after HCl hydrolysis of the resulting aqueous solution (Kobayashi et al. 1998). These last experiments showed that products were independent of the kind of irradiating particles. They showed also that the formation rate of amino acids was determined by the number of carbon monoxide molecules. Mixtures of CO and N_2_ over liquid H_2_O, irradiated with X-rays, led also to amino acids after freeze drying and HCl hydrolysis of the product aqueous solution (Takahashi et al. 1999). Mixtures of CO and NH_3_ over liquid water irradiated with protons also led to amino acids after HCl hydrolysis of the irradiation products (Takano et al. 2004a). Asymmetric syntheses of amino acid precursors have also been performed after proton irradiation of a CO, NH_3_, H_2_O mixture, followed by irradiation with right and left ultraviolet circularly polarized light (Takano et al. 2007). None of the above cited experiments gave information on the morphology of the synthesized compounds.

Envisioning a laboratory synthesis of amino acids as a consequence of the process of serpentinization, with as reactant a solid phase such as mafic or ultramafic rocks or their iron mineral constituents, olivine and pyroxenes (Bassez 2008a, b, 2009), we first irradiated with protons, a gaseous mixture of CO, N_2_ and water and we analysed the 3D-morphology of the products. We choosed CO instead of CO_2_ since earlier experiments irradiating with protons mixtures of CO_2_, N_2_ and H_2_O did not produce amino acids (Kobayashi et al. 1989, 1998). And also, we considered that CO_2_ may be transformed into CO in a natural hydrothermal process of serpentinization (Seewald et al. 2006). Discussions of abiotic synthesis of organic molecules in hydrothermal systems have focused mainly on methane and hydrocarbons (Foustoukos and Seyfried 2004; McCollom and Seewald 2007). The abiotic synthesis of amino-acids in hydrothermal systems has been suggested but is not yet demonstrated.

Here we analyse for the first time the 3D-morphology and the chirality of the products synthesized during proton irradiation of a gaseous mixture of CO, N_2_ and H_2_O. We observe filamentous and spherical micro and sub-micrometer structures which produce amino acids after HCl hydrolysis. As criteria to differentiate abiotic synthesis from contamination of biogenic origin, we used the concept of chirality and we proceeded to enantiomer analysis after derivatization of the hydrolyzed product. We observed a racemic mixture of the most abundant chiral amino acid synthesized in this study D,L-alanine, thus eliminating a biogenic contamination. Considering geology with the presence of mafic and ultramafic ferromagnesian rocks, hydrothermal chemistry with the exothermic natural process of serpentinization and the release of H_2_, the high abundance of atmospheric CO_2_, energy arising from cosmic protons or cosmic gamma rays irradiating water or cosmic radiation components, we propose that these laboratory organic microstructures may have been synthesized during Archaean Eon. The results and discussions written in the present article have been posted on Nature Precedings on 21 July 2010 (Bassez and Takano 2010). A new version considering the Earth magnetic field has been presented on a poster at the ORIGINS conference in Montpellier in July 2011 and posted on Nature Precedings on 14 November 2011 (Bassez et al. 2011).

## Materials and Method

Proton irradiation (3 MeV) was performed for 2 h, at the Tokyo Institute of Technology using a Van de Graaff accelerator. The quantity of electricity for single irradiation run was 2 mC. A Pyrex glass tube was filled with inorganic gas components consisting of 350 Torr carbon monoxide (CO) and 350 Torr nitrogen (N_2_) over 5 mL of distilled liquid water (H_2_O) which provided 20 Torr of water vapor at room temperature.

Ultra-pure grade carbon monoxide and dinitrogen gases were purchased from Nihon Sanso Co.. All glassware was heated in a high temperature oven (DR-22, Yamato Co., Tokyo, Japan) at 500 °C to eliminate any possible contaminants prior to use. Deionized water was further purified with a Millipore Milli-Q LaboSystem™ and a Millipore Simpli Lab-UV (Japan Millipore Ltd., Tokyo, Japan) to remove inorganic ions and organic contaminants.

The irradiation product analysis was conducted in the Institute of Biogeosciences, Japan Agency for Marine-Earth Science and Technology, JAMSTEC, in Yokosuka. After the surface polishing of sample plate for hydrophilic treatment by HDT-400 (JEOL), an aliquot of the unfiltered solution containing the irradiation products was gently dropped and dried at ambient temperature and ambient pressure in clean bench to obtain involatile organic matter. Morphological analysis was performed with scanning electron microscopy (SEM, JSM-6700F, JEOL: accelerating voltage < 5 kV) under low pressure (<10^−4^ Pa). Additionally, we also conducted atomic force microscopy (AFM, Seico Instruments Inc., SII SPA 400 unit, Japan) by the non-contact mode. The gel filtration chromatograph (GFC) was composed of a high performance liquid chromatography (HPLC) pump (TOSOH DP-8020) and a UV detector (TOSOH UV-8020). The separation columns used were TSKgel G2000 SWxL (7.8 mm i.d. × 300 mm) for gel filtration, and Inertsil ODS-3 (4.6 mm i.d. × 250 mm) for reversed-phase chromatography (Takano et al. 2004a). The mobile phase was a mixture of 25 mM acetonitrile (25 %) and 0.1 % trifluoroacetic acid (75 %). Molecular weights were calibrated using several molecular weights of polyethylene glycol (PEG) and human serum albumin (Takano et al. 2004a). The aqueous solution containing the irradiation products was not filtered and an aliquot was hydrolyzed with 6 M HCl at 110 °C for 24 h. Amino acids in the hydrolyzed fraction were analysed with an ion-exchanged HPLC system with analytical methods improved since the analysis of lunar samples (Kvenvolden et al. 1970; Kobayashi et al. 1990; Botta and Bada 2002; Takano et al. 2004a, b). The HPLC system used was composed of two high performance liquid chromatograph pumps (Shimadzu LC-10A), a cation exchange column (Shimpak ISC-07/S1504, 4 mm i.d. × 150 mm), a post-column derivatization system with o-phthalaldehyde and N-acetyl-L-cystein, and a Shimadzu RF-535 fluorometric detector (Takano et al. 2004b).

We also proceeded to enantiomer analysis after derivatization procedures to yield N-pivaloyl-(*S*)-2-butyl esters (NP/*S*2Bu) of the amino acid diastereoisomers (Takano et al. 2009). The NP/*S*2Bu esters were identified by a gas chromatograph/mass spectrometry (GC/MS; Agilent Technologies 6890N/5973MSD). The capillary column used for GC was an HP-5 ms (30 m × 0.32 mm i.d., 0.52 μm film thickness; Agilent Technologies). The GC oven temperature was programmed as follows: initial temperature 40 °C for 4 min, ramped up at 10 °C min^−1^ to 90 °C, and ramped up at 5 °C min^–1^ to 220 °C, where it was maintained for 10 min. The MS was scanned over *m/z* of 50–550 with the electron-impact mode set at 70 eV.

In order to obtain the yield of amino acids, we used the G-value (the number of formed molecules per 100 eV) of glycine in the hydrolyzed products, because (i) glycine is the most abundant amino acid and (ii) it was demonstrated that glycine was formed in proportion to total energy deposit including particle and photon irradiation. Discussions of G-values as a function of cosmic rays energy can be found in Kobayashi et al. 1998.

## Results

**Fig. 1:**
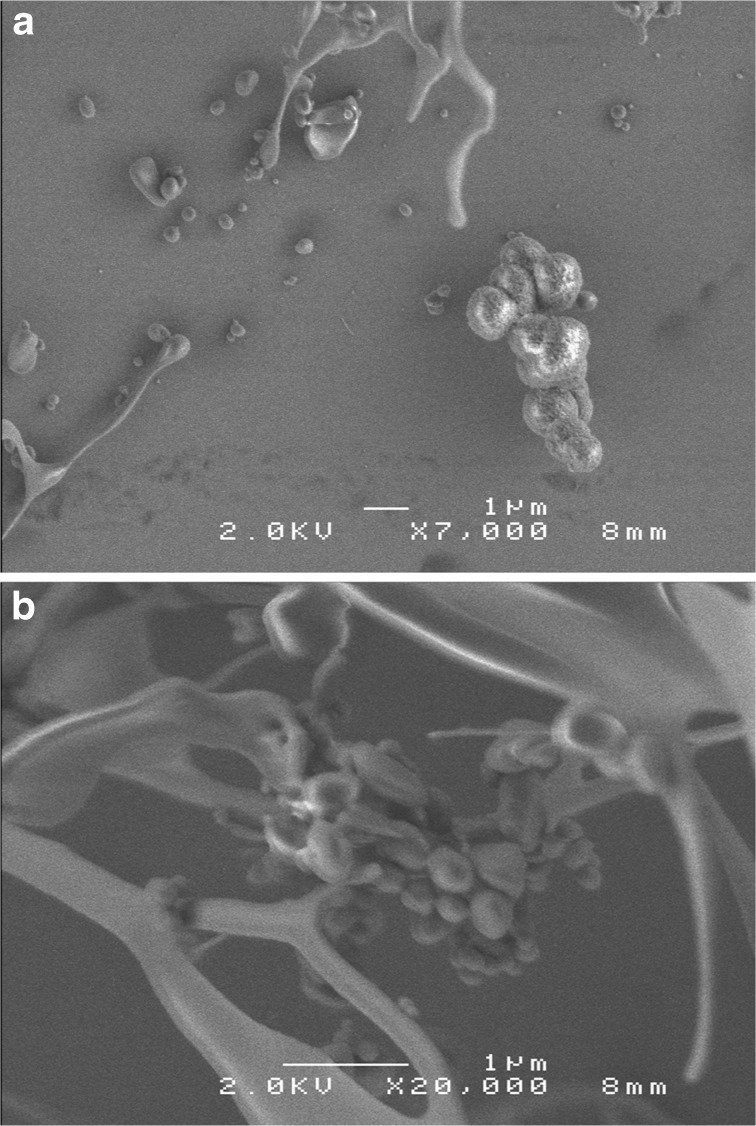
**a** Three-Dimensional Scanning Electron Microscopy, 3D-SEM, images of the dried product, abiotically synthesized from a gas mixture of CO-N_2_-H_2_O excited with 3 MeV proton irradiation; bar is 1 μm, acceleration voltage 2.0 kV, magnification ×7,000, working distance 8 mm. **b** 3D-SEM, image of the dried proton irradiation product; bar is 1 μm, acceleration voltage 2.0 kV, magnification ×20,000, working distance 8 mm

**Fig. 2:**
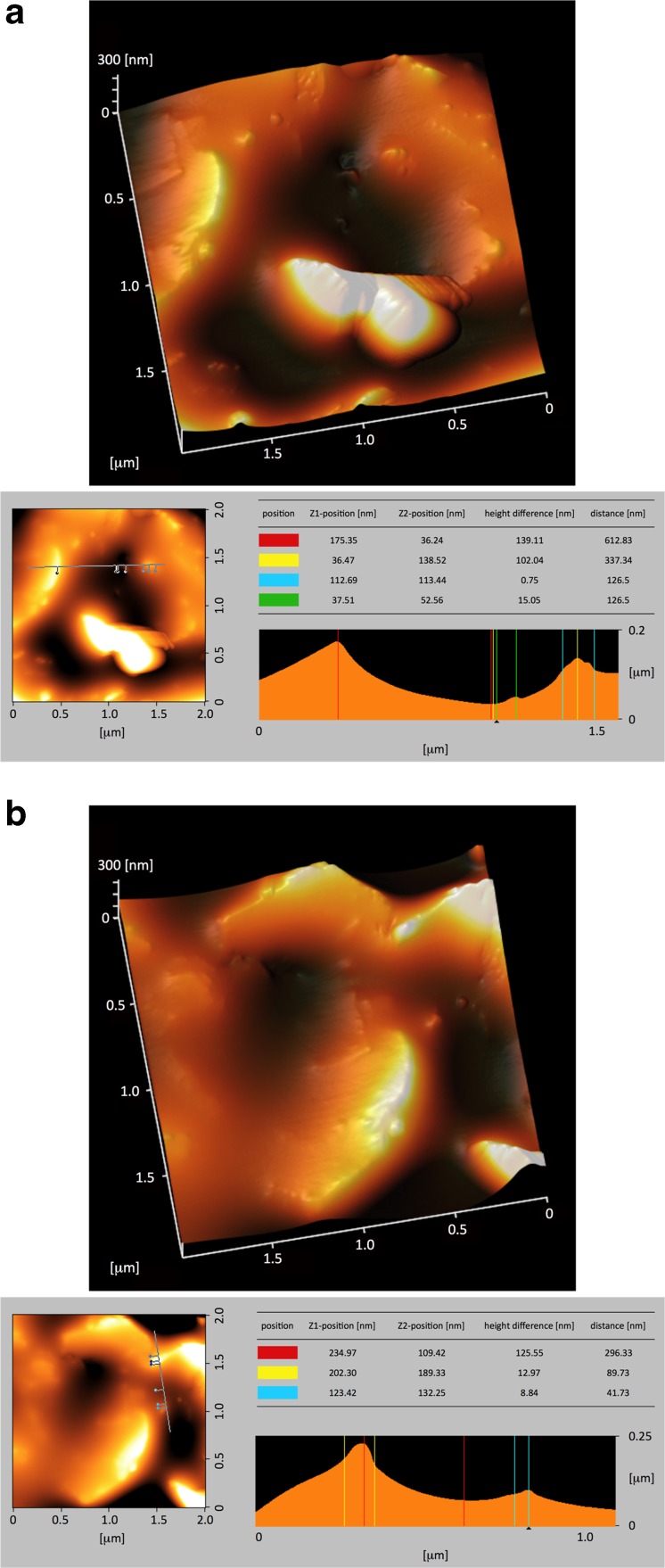
**a** 3D-Atomic Force Microscopy, 3D-AFM, images of the dried product, abiotically synthesized from a gas mixture of CO-N_2_-H_2_O, excited with 3 MeV proton irradiation. **b** 3D-AFM images of the same structure

**Fig. 3:**
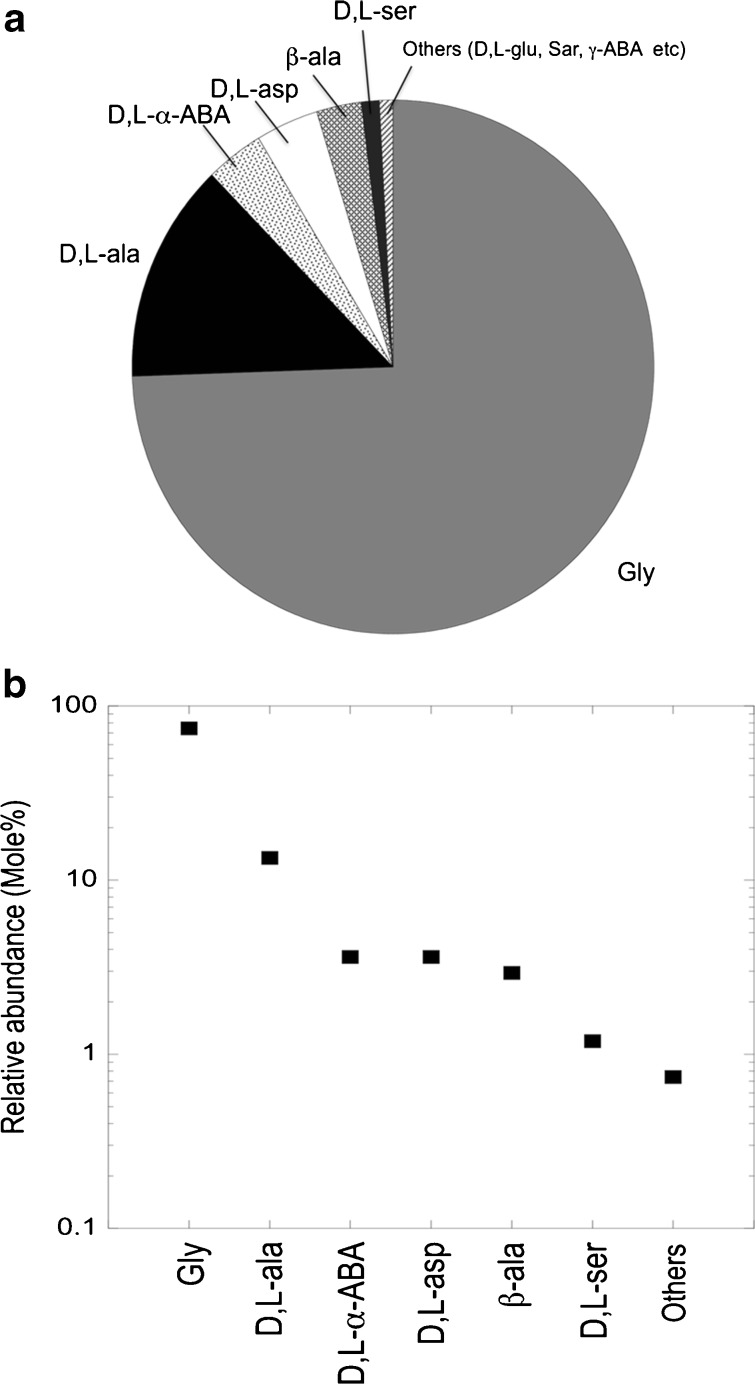
**a** Relative abundance of amino acids detected after acid hydrolysis of the dried irradiation product. Abbreviations. Gly, glycine; D,L-ala, D,L-alanine; D,L-α-ABA, D,L-α-aminobutyric acid; D,L-asp, D,L-aspartic acid; β-ala, β-alanine; D,L ser, D,L-serine; others, including very minor amino acids. **b** Relative abundance of amino acids on a logarithmic scale

**Fig. 4:**
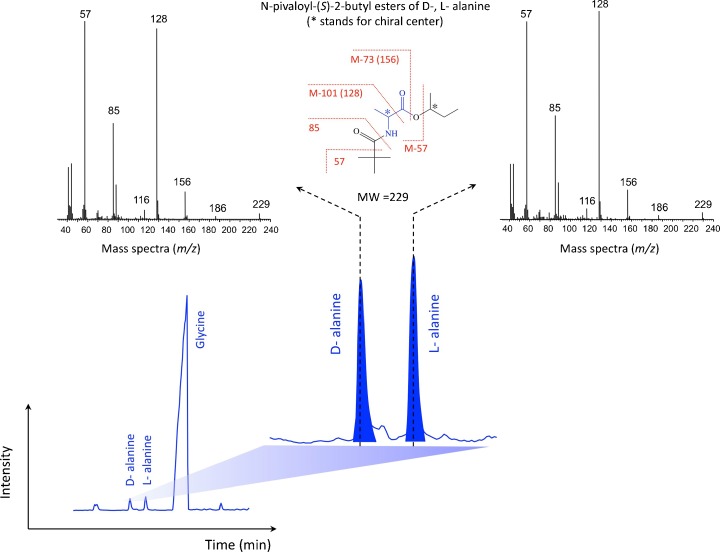
Gas chromatograph (GC) separation and its mass fragment pattern of the N-pivaloyl-(*S*)-2-butyl esters of D,L-alanine

SEM (Fig. 1a, b) and AFM (Fig. 2a, b) were performed to observe three-dimensional morphological characteristics of the yellow-colored microstructures synthesized during the irradiation. SEM images show micro- and sub-micrometer spheres, tubules and fiber-filament soft tissues. AFM was used to observe the surface of these micro- and sub-microstructures. Figure 2a and b show AFM images of the same kind of structure. On Fig. 2a is observed a depression, 140 nm in depth and 1 μm in width. On Fig. 2b is observed a depression, 125 nm in depth and 0.5 μm in width. Figure 2a shows the edges of the depression covered with protuberances which are irregular in shape. The striations observed on the white prominent parts of the depression edges (Fig. 2b) result most probably from an image of the probe tip on the depression slope and not from an image of the structure surface. However, the depression is wide enough to say that the AFM images show the surface of the structures and are not an artifact image of the probe tip. The molecular weights of these organic microstructures, determined with GFC, are distributed between several hundred and a maximum of 3000 Da. A wide variety of amino acids were detected after HCl acid-hydrolysis of this dried aliquot (Fig. 3a, b). To eliminate possible contamination results, we conducted chiral analysis after derivatization of the hydrolyzed fraction (Takano et al. 2009). Figure 4 shows GC separation of N-pivaloyl-(*S*)-2-butyl esters of D,L-alanine and glycine. The most abundant chiral amino acid, D,L-alanine, shows a racemic mixture produced by pristine abiotic chemical synthesis. Therefore, we exclude potential contamination on our organic analysis and we may conclude that the dried irradiation products are composed of abiogenic organic nano and microstructures.

It is to be noticed that we conducted earlier same analytical procedures for analyses of peridotite rocks which were dredged on the ocean floor of the mid-atlantic ridge (MAR) (Bassez et al. 2009). Non racemic mixtures of amino acids were obtained leading to the conclusion of sedimentary biological origin for the observed amino acids. These two opposite conclusions for similar analytical procedures applied to abiotic synthesis and rock analysis, also prove that our analytical procedures are exempt of biological contamination. The amino acids synthesized in this study include glycine, alanine, aspartic acid, serine and the non-proteinous amino acids β-alanine (BALA), α-aminobutyric (ABA) acid and γ-aminobutyric acid (GABA). Glycine was most abundant followed by D,L-alanine, D,L-α-aminobutyric acid, D,L-aspartic acid, β-alanine and D,L-serine, in logarithmic decrease.

The energetic yield of glycine normalized by G-value (number of synthesized molecules per 100 eV absorbed) in the present proton irradiation experiment was 0.02 (cf. Kobayashi et al. supporting data 1998).

## Discussion

Our structures are synthesized when gaseous CO and N_2_ are present over liquid water. On Earth, the source of CO could be hydrothermal, arising from the transformation of CO_2_ into CO (CO_2_ + H_2_ ↔ CO + H_2_O). The temperature of the experiment which led to the formation of CO and CH_4_ from a mixture of CO_2_ dissolved in flowing seawater, of gaseous H_2_ and of magnetite was conducted at 250 °C–300 °C and 250 bar (Fu and Seyfried 2009). Theoretical calculations showed that at 35 MPa, H_2_ production occurred during serpentinization of ultramafic rocks, between 200 and 315 °C (McCollom and Bach 2009) and that serpentinization may occur at temperatures below 300 °C (Klein and Bach 2009). H_2_ was also generated in a recent experiment conducted at 300 °C and 500 bars on hydrolysis of komatiite glass (Yoshizaki et al. 2009). At those temperatures, CO is present in both aqueous and gaseous phases. Consequently, CO is available in the gaseous phase in hydrothermal environments where olivine encounters serpentinization, producing H_2_ and magnetite. Olivine and pyroxenes minerals found in mafic and ultramafic rocks, are iron and magnesium silicates. Exothermic reactions of diverse olivine (Mg,Fe)_2_SiO_4_ and pyroxenes (Y,Fe)_x_Si_2_O_6_ with H_2_O and CO_2_ lead to products such as quartz, magnetite, serpentine, calcium carbonate, H_2_ and recently CO (Fu and Seyfried 2009). Even if the serpentinization reactions of all diverse olivine and pyroxenes have not yet been studied in detail, it is known that they are highly exothermic. Geological sites where exothermic mineral transformation occurs with a release of H_2_ are consequently appropriate sites for the transformation of CO_2_ into CO. In their environment, synthesis of abiotic organic microstructures might consecutively occur.

A recent article shows that release of H_2_ occurs at low temperature, 30 to 70 °C, when olivine containing magnetite and chromite is hydrolyzed (Neubeck et al. 2011). However at these temperatures, the formation of CO from CO_2_ is not thermodynamically favorable. Indeed, earlier experimental investigations of the CO transformation showed that substantially higher CO concentrations occur at 350 °C rather than at 150 °C (Seewald et al. 2006).

It is consequently plausible to propose that dinitrogen embedded in a ferromagnesian geological environment may react with CO and H_2_O to form molecules which may assemble during a dryness period into microspheres, filaments and tubules containing organic structures such as those synthesized in our laboratory experiment.

The abundance of CO_2_ was higher during Archaean Eon. The atmospheric partial pressure of CO_2_ was several times higher 3.2 Ga ago than present-day values (Hessler et al. 2004). The source of excitation, protons, was also higher. Protons arise from cosmic radiation or from gamma rays included in cosmic radiation which induce protons through water radiolysis. In Paleoarchaean Era, 3.5 Ga ago, the Earth magnetic field was much lower than in Phanerozoic Eon, Holocene Epoch. A very low equatorial paleointensity of ~5 μT at c.a. 3.5 Ga was reported (Hale 1987; Yoshihara and Hamano 2002) which corresponds to 17 % of the present day value. Cosmic radiation and its components could consequently easily reach the surface of the Earth. Little is known about coronal mass ejection of the Paleoarchaean Sun. However, a proton source from cosmic radiation reaching the surface of the Earth seems more probable than a proton source induced by gamma rays arising from extinct radionuclides. Indeed, the amount of radioactivity brought by the late heavy bombardment has been recently controversial. It is to be noticed that an excitation source arising from cosmic radiation, such as protons, helium nuclei and electrons would most probably produce the same kind of structures since earlier experiments (Kobayashi et al. 1998) showed that products were independent of the nature of the irradiating particles.

Experiments on the thermal alteration of these abiotic structures have been recently conducted (Kurihara et al. 2012). They show the formation of organic aggregates with aromatic carbon, at temperatures between 200 and 400 °C and under fluid pressure of 25 MPa.

## Conclusion

We demonstrate that organic micro and sub-microstructures are synthesized during proton irradiation of a gaseous mixture of CO, N_2_, H_2_O. Their shapes vary from spheres to filaments and they produce amino acids after HCl hydrolysis. The enantiomer analysis for D,L-alanine confirmed that the amino acids were abiotically synthesized during the laboratory experiment. Analysing hydrothermal, chemical and mineral conditions of natural formation on Earth, we show that these prebiotic microstructures might be synthesized during Archaean Eon, from a mixture of CO, N_2_ and H_2_O, in hydrothermal silicate environments and under an excitation source arising from cosmic radiation which existed in higher intensity 3.5 Ga ago than Phanerozoic Eon, Holocene Epoch. We show that these prebiotic microstructures might be formed as a product of the exothermic hydrolysis of the rocks and of their mineral contents during the process of serpentinization.

Amino acid precursors were first obtained from proton irradiation of CO, N_2_, H_2_O in 1989 (Kobayashi et al. 1989). Since that time, the formation of these organic molecules was discussed in primitive atmospheres rich in CO_2_ and in CO (Kobayashi et al. 1998; Miyakawa et al. 2002). Now we advance a step further, considering hydrothermal formation of CO as a product of the transformation of CO_2_ in geological sites where ferromagnesian silicate minerals encounter the process of serpentinization with the hydrothermal release of H_2_.

We suggest that a search for such organic micro and sub-microstructures, inside or nearby serpentinised rocks on Earth and on Mars, could be envisioned. The organic geochemistry of these rocks has been very little studied (Bassez et al. 2009). A discovery of such structures would confirm the hypothesis concerning prebiotic formation of amino acids near hydrothermal sites where olivine encounters serpentinization and considering a proton excitation source from cosmic radiation or as a product of water radiolysis (Bassez 2008a, b, 2009).
